# Supplementary material for: Biotic assemblages of gelatinous zooplankton in the Gulf of Mexico and adjacent waters: An evolutionary biogeographic approach
Source: PLoS One. 2024 Jul 29;19(7):e0307933. doi: 10.1371/journal.pone.0307933 (PMC11285929; doi:10.1371/journal.pone.0307933)
Supplement: S1 File — (DOCX) [file pone.0307933.s001.docx]

**1.** Ahuatzin-Hernández JM, Couoh-Concha A, Loman-Ramos L, León-Deniz LV. Range expansion of two box jellyfish (Cnidaria, Cubozoa) in southern Gulf of Mexico. Check List. 2019; 15: 405-410.

**2.** Álvarez-Cadena JN, Almaral-Mendivil AR, Ordóñez-López U, Uicab-Sabido A. Composición, abundancia y distribución de las especies de quetognatos del litoral norte del Caribe de México. Hidrobiológica. 2008; 18: 37-48.

**3.** Álvarez-Cadena JN, Suárez-Morales E, McLelland JA. Observations on an isolated population of *Sagitta hispida* Conant (Chaetognatha) in a tropical lagoon system of northeast Yucatan (Mexico). Gulf Caribb Res. 1996; 9: 197-204.

**4.** Ames C, Ohdera AH, Colston SM, Collins AG, Fitt WK, Morandini AC, et al. Fieldable environmental DNA sequencing to assess jellyfish biodiversity in nearshore waters of the Florida Keys, United States. Front Mar Sci. 2021; 8: 640527.

**5.** Barord GJ, Graham WM, Bahya KM. First report of the invasive medusa, *Phyllorhiza punctata* von Lendenfeld (1884), in Galveston Bay, Texas. Gulf Mex Sci. 2007; 25: 166-167.

**6.** Bayha KM, Graham WM, Higgins III JE, Fletcher H. Predation potential of the jellyfish *Drymonema larsoni* Bayha & Dawson (Scyphozoa: Drymonematidae) on the moon jellyfish *Aurelia* sp. in the northern Gulf of Mexico. Hydrobiologia. 2012; 690: 189-197.

**7.** Becerra-Amezcua MP, Rincón-Guevara MA, Hernández-Calderas I, Guzmán-García X, Guerrero-Legarreta I, González-Márquez H. Metalloproteinases and NAD (P) H-dependent oxidoreductase within of Bay nettle (*Chrysaora chesapeakei*) venom. Toxin Rev. 2022; 41: 280-289.

**8.** Bolton TF, Graham WM. Morphological variation among populations of an invasive jellyfish. Mar Ecol Prog Ser. 2004; 278: 125-139.

**9.** Burke WD. Biology and distribution of the macrocoelenterates of Mississippi Sound and adjacent waters. Gulf Caribb Res. 1976; 5: 17-28.

**10.** Burke WD. Pelagic Cnidaria of Mississippi Sound and adjacent waters. Gulf Caribb Res. 1975; 5: 23-38.

**11.** Canché-Canché VE, Castellanos-Osorio I. Medusae (Cnidaria) of Bahia de la Ascension, Quintana Roo, Mexico (1997). Hidrobiológica. 2005; 15: 65-72.

**12.** Castellanos-Osorio IA. Appendicularians (Tunicata) of Banco Chinchorro, Caribbean Sea. Bull Mar Sci. 2003; 73: 133-140.

**13.** De la Cruz-Francisco V, López-Torres CK, Argüelles-Jiménez J. Primer registro de *Bolinopsis vitrea* (Ctenophora: Tentaculata) para el golfo de México. Novit Caribaea. 2023; 21: 62-68.

**14.** Diupotex-Chong ME, Ocaña-Luna A, Sánchez-Ramírez M. Chromosome analysis of *Aurelia aurita* Linne, 1758 (Scyphozoa: Ulmaridae), southern Gulf of Mexico. Mar Biol Res. 2009; 5: 399-403.

**15.** Estes AM, Reynolds BS, Moss AG. *Trichodina ctenophorii* n. sp., a novel symbiont of ctenophores of the northern coast of the Gulf of Mexico. J Eukaryot Microbiol. 1997; 44: 420-426

**16.** Félix-Torres FJ, Garrido-Mora A, Sánchez-Alcudia Y, Sánchez-Martínez AJ, Granados-Berber AA, Ramos-Palma JL. Spatial and temporal abundance and distribution of *Stomolophus meleagris* (Rhizostomae: Stomolophidae) in a lagoon system Southern Gulf of Mexico. Rev Biol Trop. 2017; 65: 167-179.

**17.** Flores-Coto C, Sanvicente-Añorve L, Sánchez-Ramírez M. Distribución y diversidad de apendicularias en el sur del golfo de México. Rev Mex Biodivers. 2010; 81: 123-131.

**18.** Flores-Coto C, Sanvicente-Añorve L, Vázquez-Gutiérrez F, Sánchez-Ramírez M. Mesoscale distribution of Oikopleura and Fritillaria (Appendicularia) in the Southern Gulf of Mexico: spatial segregation. Rev Biol Mar Oceanogr. 2010; 45: 379-388.

**19.** Flores-Coto C. Contribución al conocimiento de las apendicularias del arrecife “La Blanquilla” Veracruz, México, con descripción de una nueva especie. Anales del Centro de Ciencias del Mar y Limnología, Universidad Nacional Autónoma de México. 1974; 1: 41-60.

**20.** Flores-Galicia L, De la Cruz-Francisco V. Primeros registros de medusas (Cubozoa, Scyphozoa), sifonóforos (Hydrozoa) y ctenóforos (Tentaculata) del sistema arrecifal Lobos-Tuxpan, México. Investig Mar CICIMAR. 2018; 33: 25-38.

**21.** Frolova A, Muffett K, Miglietta MP. Multiple occurrences of *Callinectes sapidus* larvae on Gulf of Mexico *Chrysaora chesapeakei*. J Plankton Res. 2022; 44: 966-969.

**22.** Frost JR, Jacoby CA, Frazer TK, Zimmerman AR. Pulse perturbations from bacterial decomposition of *Chrysaora quinquecirrha* (Scyphozoa: Pelagiidae). Hydrobiologia. 2012; 690: 247-256.

**23.** Gilmartin J, Yang Q, Liu H. Seasonal abundance and distribution of chaetognaths in the northern Gulf of Mexico: The effects of the Loop Current and Mississippi River plume. Cont Shelf Res. 2020; 203: 104146.

**24.** Gómez-Aguirre S. Variación estacional de grandes medusas (Scyphozoa) en un sistema de lagunas costeras del sur del golfo de México (1977/1978). Boletim do Instituto Oceanográfico. 1980; 29: 183–185.

**25.** Graham WM, Martin DL, Felder DL, Asper VL, Perry HM. Ecological and economic implications of a tropical jellyfish invader in the Gulf of Mexico. Biol Invasions. 2003; 5: 53-69.

**26.** Graham WM. First report of *Carybdea alata* var. *grandis* (Reynaud 1830) (Cnidaria: Cubozoa) from the Gulf of Mexico. Gulf Mex Sci. 1998; 16: 28-30.

**27.** Guest WC. The occurrence of the jellyfish *Chiropsalmus quadrumanus* in Matagorda Bay, Texas. Bull Mar Sci. 1959; 9: 79-83.

**28.** Harper Jr DE, Runnels RJ. The occurrence of *Rhopilema verrilli* (Cnidaria: Scyphozoa: Rhizostomeae) on Galveston Island, Texas, and a discussion on its distribution in U.S. Waters. Gulf Mex Sci. 1990; 11:19-27.

**29.** Hernández-Flores RM, Suárez-Morales E, Gasca R. Seasonal distribution of Chaetognatha in a Mexican neotropical bay during a year cycle. J Coast Res. 2005; 21: 186-192.

**30.** Hernández-Flores RM. Report on a collection of chaetognaths from Banco Chinchorro, Mexican Caribbean Sea. Bull Mar Sci. 2003; 73: 123-131.

**31.** Kellner K. On *Oikopleura tortugensis*, a new appendicularian from the Tortugas, Florida, with notes on its embryology. In: Brooks WK, editor. The pelagic Tunicata of the Gulf Stream. Washington: Carnegie Inst.; 1908. pp. 89-94.

**32.** Larson RJ. Diet, prey selection and daily ration of *Stomolophus meleagris*, a filter-feeding scyphomedusa from the NE Gulf of Mexico. Estuar Coast Shelf Sci. 1991; 32: 511-525.

**33.** Lasley Jr RM, Ames CL, Erdman R, Parks S, Collins AG. First record of the box jellyfish *Tripedalia cystophora* (Cnidaria: Cubozoa: Tripedaliidae) in the Gulf of Mexico. Proc Biol Soc Wash. 2016; 129: 164-172.

**34.** Lazcano-Pérez F, Arellano RO, Garay E, Arreguín-Espinosa R, Sánchez-Rodríguez J. Electrophysiological activity of a neurotoxic fraction from the venom of box jellyfish *Carybdea marsupialis*. Comp Biochem Physiol C Toxicol Pharmacol. 2017; 191: 177-182.

**35.** Loman-Ramos L, Ordóñez-López U, Segura-Puertas L. Variación espacial de la comunidad de medusas (Cnidaria) del sur del Golfo de México, durante el otoño de 1999. Hidrobiológica. 2007; 17: 203-212.

**36.** López-Torres CK, Mendoza-Becerril MA, de la Cruz-Francisco V. Medusozoans of Tuxpan, Veracruz, Gulf of Mexico. Reg Stud Mar Sci. 2023; 63: 102987.

**37.** Lozano-Cobo H, del Prado-Rosas MCG, Sánchez-Velasco L, Gómez-Gutiérrez J. Seasonal variation in chaetognath and parasite species assemblages along the northeastern coast of the Yucatan Peninsula. Dis Aquat Organ. 2017; 124: 55-75.

**38.** Lucas CH, Reed AJ. Gonad morphology and gametogenesis in the deep-sea jellyfish *Atolla wyvillei* and *Periphylla periphylla* (Scyphozoa: Coronatae) collected from Cape Hatteras and the Gulf of Mexico. J Mar Biol Assoc U K. 2010; 90: 1095-1104.

**39.** Martell-Hernández LF, Sánchez-Ramírez M, Ocaña-Luna A. Distribution of planktonic cnidarian assemblages in the southern Gulf of Mexico, during autumn. Rev Chil Hist Nat. 2014; 87: 18.

**40.** McLelland JA, Perry HM. Records of deep-water chaetognaths from the Northern Gulf of Mexico. Gulf Caribb Res. 1989; 8: 181-187.

**41.** McLelland JA. An illustrated key to the Chaetognatha of the northern Gulf of Mexico with notes on their distribution. Gulf Caribb Res. 1989; 8: 145-172.

**42.** McLelland JA. *Eukrohnia calliops*, a new species of Chaetognatha from the northern Gulf of Mexico with notes on related species. Proc Biol Soc Wash. 1989; 102: 33-44.

**43.** McLelland JA. Observations on chaetognath distributions in the northeastern Gulf of Mexico during the summer of 1974. Gulf Mex Sci. 1984; 7: 49-59.

**44.** Mecalco-Hernández Á, Castillo-Rivera M. Riqueza zooplanctónica en la boca de la Laguna La Mancha, durante muestreos mensuales y nictímeros. Hidrobiológica. 2020; 30: 143-153.

**45.** Mille-Pagaza S, Carrillo-Laguna J. Distribución y abundancia de los quetognatos de la plataforma Tamaulipeca y océano adyacente en abril de 1987. Hidrobiológica. 2003; 13: 223-229.

**46.** Mille-Pagaza S, Carrillo-Laguna J. Los quetognatos (Chaetognatha) del banco de Campeche en abril-mayo de 1986. Rev Biol Trop. 1999; 47: 101-108.

**47.** Mille-Pagaza S, Carrillo-Laguna J. The Chaetognatha of the southwestern Gulf of Mexico during April-May, 1986. Gulf Caribb Res. 2001; 13: 51-57.

**48.** Mille-Pagaza S, Reyes-Martinez R, Sanchez-Salazar ME. Distribution and abundance of Chaetognatha on the Yucatan Shelf during May, 1986. Gulf Caribb Res. 1997; 9: 263-275.

**49.** Moore DR. The occurrence of *Stephanoscyphus corniformis* Komai (Scyphozoa) in the western Atlantic. Bull Mar Sci. 1961; 11: 319-320.

**50.** Mulkana MS, McIlwain TD. The seasonal occurrence and abundance of Chaetognatha in Mississippi Sound. Gulf Caribb Res. 1973; 4: 264-271.

**51.** Ocaña-Luna A, Mecalco-Hernández Á, Sánchez-Ramírez M, Castillo-Rivera M. Nuevos registros y morfometría de *Pleurobrachia pileus* (Phylum Ctenophora) en el golfo de México. Rev Mex Biodivers. 2017; 88: 442-445.

**52.** Ocaña-Luna A, Sánchez-Ramírez M, Aguilar-Durán R. First record of *Phyllorhiza punctata* von Lendenfeld, 1884 (Cnidaria: Scyphozoa, Mastigiidae) in Mexico. Aquat Invasions. 2010; 5: S79-S84.

**53.** Ocaña-Luna A, Sánchez-Ramírez M, Aguilar-Durán R. Macromedusas y ctenóforos del sistema arrecifal veracruzano y lagunas costeras asociadas. In: Granados-Barba A, Ortiz-Lozano L, Salas-Monreal D, González-Gándara C, editors. Aportes al conocimiento del sistema arrecifal veracruzano: Hacia el corredor arrecifal del suroeste del Golfo de México. Campeche: Universidad Autónoma de Campeche; 2015. pp. 121-138.

**54.** Orellana ER, Collins AG. First report of the box jellyfish *Tripedalia cystophora* (Cubozoa: Tripedaliidae) in the continental USA, from Lake Wyman, Boca Raton, Florida. Mar Biodivers Rec. 2011; 4: e54.

**55.** Owre HB. A new chaetognath genus and species, with remarks on the taxonomy and distribution of others. Bull Mar Sci. 1973; 23: 948-963.

**56.** Owre HB. Plankton of the Florida current. Part VI. The chaetognatha. Bull Mar Sci. 1960; 10: 255-322.

**57.** Owre HB. Some temperatures, salinities, and depths of collection of *Eukrohnia bathyantarctica* (Chaetognatha) in the Caribbean Sea. Bull Mar Sci. 1972; 22: 94-99.

**58.** Phillips PJ, Burke WD. The occurrence of sea wasps (Cubomedusae) in Mississippi Sound and the northern Gulf of Mexico. Bull Mar Sci. 1970; 20: 853-859.

**59.** Phillips PJ, Levin NL. Cestode larvae from scyphomedusae of the Gulf of Mexico. Bull Mar Sci. 1973; 23: 574-584.

**60.** Phillips PJ. The occurrence of the remarkable scyphozoan, *Deepstaria enigmatica*, in the Gulf of Mexico and some observations on cnidarian symbionts. Gulf Caribb Res. 1973; 4: 166-168.

**61.** Puente-Tapia FA, Espinosa-Fuentes ML, Zavala-García F, Olguín-Jacobson C, Flores-Coto C. Spatial distribution of medusae (Cnidaria) assemblages in the southern Gulf of Mexico (dry season). Community Ecol. 2022; 23: 137-162.

**62.** Puente-Tapia FA, Gasca R, Schiariti A, Haddock SH. An updated checklist of ctenophores (Ctenophora: Nuda and Tentaculata) of Mexican seas. Reg Stud Mar Sci. 2021; 41: 101555.

**63.** Puente-Tapia FA, Ortigosa D, Vital XG, Palomino-Alvarez L A. First record of a benthic ctenophore in the Mexican Atlantic: *Vallicula multiformis* (Platyctenida, Coeloplanidae). J Mar Biol Assoc U K. 2023; 103: e47.

**64.** Ramírez-Avila Y, Álvarez-Cadena JN. Chaetognath species composition from a coral reef lagoon in the Mexican Caribbean Sea. Rev Biol Trop. 1999; 47: 157-163.

**65.** Ramos G, Segura-Puertas L. Seasonal occurrence of reef-related medusae (Cnidaria) in the Western Caribbean Sea. Gulf Caribb Res. 2004; 16: 1-9.

**66.** Roden CL, Lohoefener RR, Rogers CM, Mullin KD, Hoggard BW. Aspects of the ecology of the moon jellyfish, *Aurelia aurita*, in the northern Gulf of Mexico. Gulf Mex Sci. 1990; 11: 63-67.

**67.** Sánchez-Rodríguez J, Lucio-Martínez NL. Isolation and prepurification of active compounds in venom from *Pelagia noctiluca* (Scyphozoa: Pelagiidae) from the Caribbean Sea. Cienc Mar. 2011; 37: 369-377.

**68.** Sanvicente-Anorve L, Sierra-Zapata S, Lemus-Santana E, Ruiz-Boijseauneau I, Soto LA. Feeding of *Flaccisagitta enflata* (Chaetognatha) upon copepods in the southern Gulf of Mexico. Cah Biol Mar. 2020; 61: 1-7.

**69.** Suárez-Caabro JA, Madruga JE. The Chaetognatha of the northeastern coast of Honduras, Central America. Bull Mar Sci. 1960; 10: 421-429.

**70.** Suárez-Morales E, Segura-Puertas L, Gasca R. Medusan (Cnidaria) assemblages off the Caribbean coast of Mexico. J Coast Res. 1999; 15: 140-147.

**71.** Tokioka T. Notes on some chaetognaths from the Gulf of Mexico. Bull Mar Sci. 1955; 5: 52-65.

**72.** Tovar E, Suárez-Morales E, Carrillo L. Multiscale variability of the Chaetognatha along a Caribbean reef lagoon system. Mar Ecol Prog Ser. 2009; 375: 151-160.

**73.** Tovar E, Suárez-Morales E. New records and a new species of *Spadella* (Chaetognatha: Spadellidae) from the western Caribbean Sea. Proc Biol Soc Wash. 2007; 120: 175-183.

**74.** Tunberg BG, Reed SA. Mass occurrence of the jellyfish *Stomolophus meleagris* and an associated spider crab *Libinia dubia*, Eastern Florida. Florida Scientist. 2004; 67: 93-104.

**75**. Winkler JT, van Soest R. First record of the scyphomedusa *Deepstaria enigmatica* Russell, 1967, from the mid North Atlantic Ocean (Coelenterata, Scyphozoa). Beaufortia. 1981; 8: 33-37.

**76.** Witmer AD, Chesnes T, Miller Z, Tedford S, Bell AC. New records of *Tripedalia cystophora* Conant, 1897 (Cubozoa, Carybdeida) along the southeastern Florida coastline. Check List. 2023; 19: 57-62.
